# Functional Analysis of Sirtuin Genes in Multiple *Plasmodium falciparum* Strains

**DOI:** 10.1371/journal.pone.0118865

**Published:** 2015-03-17

**Authors:** Catherine J. Merrick, Rays H. Y. Jiang, Kristen M. Skillman, Upeka Samarakoon, Rachel M. Moore, Ron Dzikowski, Michael T. Ferdig, Manoj T. Duraisingh

**Affiliations:** 1 Department of Immunology & Infectious Diseases, Harvard School of Public Health, Boston, Massachusetts, United States of America; 2 Department of Biological Sciences, Eck Institute for Global Health, University of Notre Dame, Notre Dame, Indiana, United States of America; 3 Department of Microbiology & Molecular Genetics, The Kuvin Center for the Study of Infectious and Tropical Diseases, IMRIC, The Hebrew University-Hadassah Medical School, Jerusalem, Israel; Bernhard Nocht Institute for Tropical Medicine, GERMANY

## Abstract

*Plasmodium falciparum*, the causative agent of severe human malaria, employs antigenic variation to avoid host immunity. Antigenic variation is achieved by transcriptional switching amongst polymorphic *var* genes, enforced by epigenetic modification of chromatin. The histone-modifying ‘sirtuin’ enzymes PfSir2a and PfSir2b have been implicated in this process. Disparate patterns of *var* expression have been reported in patient isolates as well as in cultured strains. We examined *var* expression in three commonly used laboratory strains (3D7, NF54 and FCR-3) in parallel. NF54 parasites express significantly lower levels of *var* genes compared to 3D7, despite the fact that 3D7 was originally a clone of the NF54 strain. To investigate whether this was linked to the expression of sirtuins, genetic disruption of both sirtuins was attempted in all three strains. No dramatic changes in *var* gene expression occurred in NF54 or FCR-3 following PfSir2b disruption, contrasting with previous observations in 3D7. In 3D7, complementation of the PfSir2a genetic disruption resulted in a significant decrease in previously-elevated *var* gene expression levels, but with the continued expression of multiple *var* genes. Finally, rearranged chromosomes were observed in the 3D7 PfSir2a knockout line. Our results focus on the potential for parasite genetic background to contribute to sirtuin function in regulating virulence gene expression and suggest a potential role for sirtuins in maintaining genome integrity.

## Introduction

Malaria caused by *Plasmodium falciparum* gives rise to widespread morbidity and approximately a million deaths each year. During its asexual replicative lifecycle, the parasite lives inside human erythrocytes and is spread between hosts via mosquito bite. The parasite can maximize transmission by avoiding the human immune system and sustaining chronic infections; consequently, *P*. *falciparum* has evolved a complex system of antigenic variation, allowing infections to persist for months or years.

Antigenic variation is best characterized for the major surface antigen expressed on infected erythrocytes, PfEMP1 [1–4]. This large transmembrane protein mediates adhesion to a variety of host endothelial molecules, sequestering infected cells out of the circulation to avoid splenic clearance. The exposed PfEMP1 protein is thus a target for host immunity [5] and a large family of variant ‘*var*’ genes encoding PfEMP1 has evolved. There are 63 *var* genes in the sequenced 3D7 strain, with the majority located sub-telomerically and a subset arranged in tandem arrays at chromosome-internal locations [6]. The sub-telomeric genes are divided into groups termed upsA and upsB according to similarities in upstream sequence and gene orientation. There is also a unique sub-telomeric ‘upsE’ gene. Chromosome-internal genes, meanwhile, belong to a separate ‘upsC’ group. Several studies have linked high expression of sub-telomeric *var* genes to severe forms of malaria, showing that the upsA and/or upsB genes are expressed in children with severe disease [7–11] and the upsE gene, in pregnancy related malaria [12].

The majority of *var* genes are silenced at any one time, with periodic switching of active gene(s). Although *var* genes appear to be expressed in a mutually exclusive fashion [13–16], a more relaxed transcriptional pattern has been described in the 3D7 parasite strain. 3D7 expresses several *var* genes at significant levels [17–20] and represents these simultaneously as protein on the cell surface [20], whereas NF54 shows tight allelic exclusion [21].

The mechanism that controls *var* silencing, activation and switching appears to be epigenetic because expression patterns are maintained semi-stably for multiple generations [22] and because *var* family silencing is disrupted by mutation of either of the two *P*. *falciparum* sirtuin genes [4,23]. Sirtuins are NAD^+^-dependent histone deacetylases that facilitate chromatin condensation and heterochromatic gene silencing in model systems. The larger of the two *P*. *falciparum* sirtuins, PfSir2b, remains biochemically uncharacterized but the smaller, PfSir2a, is a histone deacetylase with a role in chromatin condensation and *var* gene silencing [24,25]. Initial studies conducted in 3D7 showed that the loss of *PfSir2a* causes de-silencing of multiple *var* genes, particularly the sub-telomeric upsA, BA and E groups [23], while the loss of *PfSir2b* causes a modest loss of silencing amongst upsB genes [4].

Sirtuins in model organisms often act specifically at telomeres. The term ‘telomere position effect’ (TPE) refers to a gradient of sirtuin-dependent heterochromatic silencing that extends into chromosomes from telomeres in budding yeast [26]. The *PfSir2a* and *PfSir2b* mutant phenotypes suggest that TPE also exists in *P*. *falciparum*. Furthermore, PfSir2a binds to sub-telomeric chromatin [25] and may have a direct effect on telomere maintenance, since telomeres are dramatically elongated in the absence of PfSir2a [4]. Consistently, a mammalian sirtuin, SIRT1, has been shown to affect telomere maintenance in mouse cells [27], as well as having complex effects upon DNA repair and genomic integrity [28].

Despite accumulating evidence that the sirtuins affect both virulence gene expression and telomere maintenance in *P*. *falciparum*, some important questions remain unanswered. Firstly, TPE may not account for the entire mechanism of *var* gene silencing. Histone modification (according to genome-wide chromatin immunoprecipitation) changes evenly throughout sub-telomeres when *PfSir2a* is mutated [29], yet not every sub-telomeric *var* gene is activated and some chromosome-central genes are also affected [30]. Therefore, the present study investigated whether PfSir2a may affect gene expression via molecular mechanisms besides TPE. Secondly, we wished to establish whether all sirtuin-related phenotypes are conserved across strain backgrounds and to evaluate whether the transcription of different *var* gene sub-types may be differently controlled by PfSir2a and PfSir2b. Thirdly, we investigated the effect of complementing PfSir2a in the 3D7 mutant. Our results reveal significant strain-to-strain differences in Sir2 phenotypes in long-term culture-adapted parasite strains, and point to a plethora of roles for sirtuins in controlling virulence gene expression, telomere maintenance and chromosomal stability.

## Materials and Methods

### Parasites and parasite culture

3D7 parasite isolates were obtained from three different sources and clonal parasites were generated from bulk culture by limiting dilution. The three sources included the MR4 strain (clones 4,5,11,14,18), the HR1.2 strain (clones 2, 10) and strain KW (gift obtained from Dr. Kim Williamson, Loyola University) (clones G10 and G11). NF54 clones were generated and analyzed from the MR4 strain (clones 21 and 23). NF54 clones were compared with data from previously generated clones of NF54 (a3, c3, g6, b3) as processed in Frank *et al*. [31]. Clones from FCR-3 were generated as described in Janes *et al*. [11].

All *P*. *falciparum* lines were cultured in human O^+^ erythrocytes obtained from Research Blood Components (Boston, MA) at 4% haematocrit in RPMI 1640 supplemented with 0.5% albumax (Invitrogen) and 0.25% sodium bicarbonate, using standard procedures [32]. Donor consent was waived by the institutional review board due to deidentification of the samples. Cloning was achieved by limiting dilution [33] and *var* expression profiles were assessed as soon as possible after cloning (15–20 generations). ~2x10^8^ ring-stage parasites were collected for all ring-stage RNA preparations: synchronized rings were produced by treatment with 5% sorbitol. These parasites were then kept in culture until schizonts were observed to collect late-stage RNA samples.

### Plasmid construction and generation of knockout parasite lines

Knockout parasites were generated using *PfSir2a* and *PfSir2b* targeting plasmids previously used and described in [4,23]. An adapted schematic for generation of these lines is presented in S3 Fig. Transfection of the *PfSir2a* and *PfSir2b* targeting plasmids into the FCR-3 and NF54 backgrounds was carried out as previously described [34]. For complementation studies, a plasmid containing the coding sequence for *PfSir2a* (PF3D7_1328800) under 1.75kb of its own upstream sequence from 3D7 was constructed in the plasmid vector pLNENRGFP [35]. The *PfSir2a* coding sequence was amplified from 3D7 genomic DNA (Sir2aF/Sir2aR) and cloned into pLNENRGFP as an AvrII/AflII fragment. The construct was verified by sequencing. The *PfSir2a* upstream sequence was then amplified (Sir2proF/Sir2proR) and cloned into this plasmid as an Apa1/AvrII fragment, replacing the calmodulin promoter sequence. Primers are described in S7 Table. Transfection of the *PfSir2a* complementation plasmid was performed as above, with selection on 2.5mg/ml Blasticidin HCl (Sigma).

### Southern blotting and pulsed field gel electrophoresis

Telomere Restriction Fragment Southern blots were performed as previously described [36]; briefly, genomic DNA was digested with AluI, DdeI, MboII and RsaI, and blotted using a probe amplified from the *P*. *falciparum* telomeric sequence. The median telomere length in each genome was measured using Telometric software. (Note that one replicate blot for 3D7, 3D7ΔSir2a and 3D7ΔSir2b was measured from Tonkin *et al*. [4].) Southern blots to confirm successful disruptions of the *PfSir2a* and *PfSir2b* genes were carried out as previously described [4,23], using the 5’ ends of the Sir2a and Sir2b genes respectively as probes. Acc1/Nde1 were used for digestion of FCR-3Δ*sir2a* and NF54Δ*sir2a* and ScaI/HpaII for FCR-3Δ*sir2b* and NF54Δ*sir2b* digestion. Southern blots to assess rearrangement or amplification of sub-telomeric repeat sequences were carried out by digesting genomic DNA with Taq1 and using the probes ‘TARE1’ and ‘ATS’ (kind gift from Dr. Ingrid Felger, Swiss Tropical Institute). Pulsed Field Gel Electrophoresis was carried out as previously described [37].

### RT-PCR for *var* gene expression

cDNAs were prepared and assessed essentially as described in [38]. Briefly, RNA was extracted from parasites using Trizol Reagent (Invitrogen), purified on PureLink columns (Invitrogen), treated with DNaseI (Invitrogen) and then reverse-transcribed using the Superscript II reverse transcriptase kit (Invitrogen). Each cDNA was checked for gDNA contamination using PCR across the intron of the gene PFD1155w, as described by Frank *et al*. [31]. RT-PCR for *var* gene expression in the 3D7 and NF54 backgrounds was carried out using the primer set designed by Salanti *et al*. [12], with modifications as in [31,38]. Primers and PCR conditions for the *var* family in FCR-3 are previously described [39].

RT-PCR was carried out using ITAQ SYBR SUPERMIX (Bio-Rad) in an ABI Prism 7500 thermocycler. Conditions for 3D7 and NF54 cDNAs are described in [38]; for FCR-3, 20 μl reactions were carried out using Power-SYBR Green Master Mix at final primer concentrations of 0.05 μM-0.5 μM under the following PCR conditions: 50°C for 1 min, 95°C for 10 min, then 40 cycles of dissociation, annealing, and extension at 95°C for 15 sec, 52°C for 15 sec, and 60°C for 45 sec, respectively.

Slight variations in primer efficiency were accounted for by the method of Dzikowski *et al*. [38] with correction factors listed in S8 Table; RT-PCR was carried out in duplicate on gDNA and a ΔCt for each primer set was then calculated relative to a housekeeping gene: PF07_0073 (seryl-tRNA synthetase) for 3D7 or adenylosuccinate lyase (ASL, PFB0295w) for FCR-3. This ‘correction factor’ was applied to all experimental ΔCt values. The gDNA analysis also confirmed NF54 clones analysed here have a 3D7-like genome not an E5-like genome [44]. Relative Copy Number (RCN) were calculated as 2^ΔCt^, where ΔCt was relative to control genes. For *var* subset analysis between strains, control genes were PF07_0073 (seryl-tRNA synthetase) for 3D7/NF54 or adenylosuccinate lyase (ASL, PFB0295w) for FCR-3. For the individual *var* gene RCN of sirtuin knockout strains, the average Ct of the 3 control genes: PF07_0073 (seryl-tRNA synthetase), PFL0900c (arginyl-tRNA synthetase), PF13_0170 (glutaminyl-tRNA synthetase) was used for all strains.

### RT-PCR for sirtuin expression

cDNAs were prepared from schizont-stage samples as described for ring-stage samples, and quantitative PCR was carried out using the primers listed in S7 Table for *PfSir2a*, *PfSir2b* and the control genes MAL13P1.148 (myosin) and PF07_0073 (seryl-tRNA synthetase). ΔΔCt analysis was carried out to yield the Relative Copy Number (RCN) of the sirtuin genes calculated as 2^ΔCt^, where ΔCt was relative to the average Ct of the 2 control genes. Experiments were carried out in both biological and technical duplicate.

### Comparative genome hybridization

A custom 385 k NimbleGen array designed for the *P*. *falciparum* 3D7 reference genome (PlasmoDB, 2006) using the standard CGH probe design protocol [40] was used [41]. The array comprises 385,585 probes semi-tiled across the genome at a 4 bp interval spacing with a minimum probe length of 45 bp, and a maximum length of 85 bp. Labeling and hybridization was carried out according to the standard NimbleGen CGH protocol [40]. Genomic DNAs (gDNA) from the NF54 and the 3D7Δ*PfSir2a* parasite lines were co-hybridized to CGH microarrays with reference 3D7. DNA fragmentation, labeling, hybridization, washing and scanning were carried out using the standard NimbleGen CGH protocol at the Genomics Core Facility (University of Notre Dame, Notre Dame, IN). The microarrays were hybridized and washed in a NimbleGen Hybridization System 4 (NimbleGen Systems, Inc.). Images were acquired by using a The NimbleGen MS 200 Microarray Scanner (NimbleGen Systems, Inc.) at a 5 μm resolution. Probe intensity values were extracted from scanned images using NimbleScan extraction software (NimbleGen Systems, Inc.). The Cy3 and Cy5 signal intensities were normalized according to standard Nimblegen protocol (http://www.nimblegen.com/products/lit/cgh_userguide_v6p0.pdf). The normalized values were used for calculation of Log_2_ratio values and used for CNV detection. The data discussed in this publication have been deposited in NCBI’s Gene Expression Omnibus [42] and are accessible through the GEO series accession number GSE61940 (http://www.ncbi.nlm.nih.gov/geo/query/acc.cgi?acc=GSE61940).

### CNV detection criteria

Each probe was blasted (NCBI BLAST 2.1.1, without low complexity filtering) against the 3D7 reference genome (PlasmoDB v5.4) and non-unique probes were discarded. A total of 383,333 probes were used for CNV analysis. Segmentation analysis for identification of CNV regions was performed with CGHSeg algorithm [43] using CGHweb (http://compbio.med.harvard.edu/CGHweb). As *P*. *falciparum* is a haploid organism, relatively low single value cutoffs of log_2_ratio of normalized Cy5/Cy3 values, 0.5 and -0.5, were used for the primary level identification of CNVs. Additionally for a region to be considered a CNV, the lists of candidate CNV regions were filtered using 2 different stringency criteria. 1) CNV regions were required to carry 10 or more probes; 2) the average log_2_ratio value of the normalized Cy5/Cy3 values of all the probes spanning a CNV region was required to be ≤ -0.8 to be considered a loss, or ≥0.8 to be considered a gain.

## Results

### Polarized expression and quantitative variation in expression of *var* gene subsets in common lab strains

To understand the expression patterns of *var* subsets in different parasite lines, we obtained three common lab strains for comparison. Since *var* expression patterns are only semi-stable with periodic switching, all parasites were cloned by limiting dilution to obtain homogenous populations prior to assessment. For each strain, clones were obtained from lines that had been separately maintained for months or years. Nine 3D7 clones were obtained from three backgrounds, including five derived from the MR4 strain, two from ‘3D7 HR1.2’, a derivative of the MR4 strain with a drug-resistance gene integrated at a non-coding sub-telomeric locus [23], and two clones from ‘3D7 KW’, a derivative selected for the inability to produce gametocytes. Similarly, clones from different sources of NF54 and FCR-3 were compared. Two NF54 clones were freshly derived from the MR4 strain and compared to previous data obtained from independently-derived NF54 clones [31]. Data from nine FCR-3 clones, previously derived from two different backgrounds, were also included in the analysis [11].

**Fig 1:**
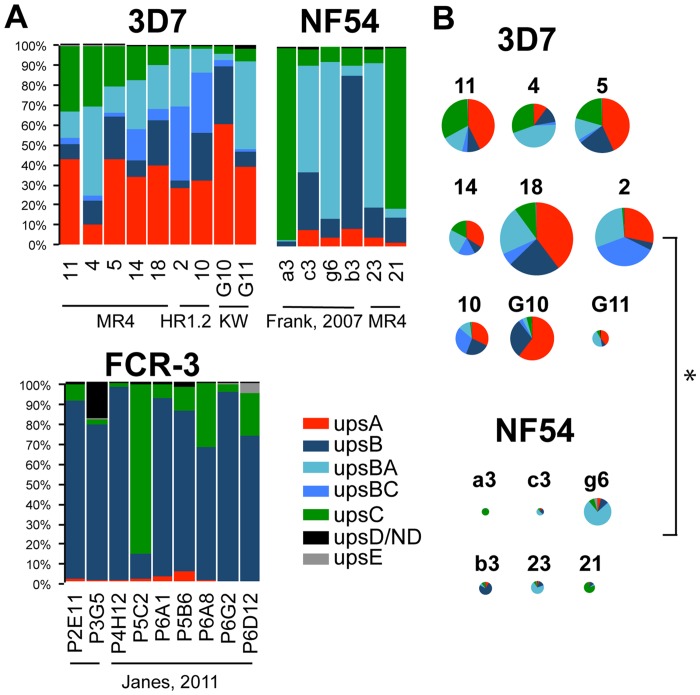
Polarized expression of *var* genes subsets in different strains. A: Analysis of *var* subset expression within clones of 3D7, NF54 and FCR-3 from disparate sources, demonstrating skews in *var* sub-class expression. 3D7 clones were derived from three different sources (MR4 3D7, 3D7 HR1.2 and 3D7 KW). NF54 clones were derived from the MR4 strain and *var* gene expression was compared to that previously obtained from clones of a distinct source of NF54 [31]. *Var* expression data for FCR-3 was obtained from clones previously examined from two parental parasite sources [11]. *Var* expression patterns are group representations that were determined by qRT-PCR using gene-specific primers for the entire *var* family in 3D7/NF54, normalized to seryl-tRNA-synthetase and sorted by ups-group. *Var* expression patterns were measured by qRT-PCR using gene-specific primers for the majority of the family in FCR-3, sorted by ups-group and normalized to the average abundance of the transcript of adenylosuccinate lyase. Independent clones are shown for each strain (different sources are denoted by underline). B: Relative copy numbers (RCN) for all of the genes within each 3D7 and NF54 strain were summed, and the proportions of expression of each subset within each genetic background are indicated. RCN was calculated by comparison to the abundance of transcripts encoding seryl-tRNA-synthetase (results were also similar if compared to the ring-stage-specific genes encoding SBP1 (PF3D7_0501300) and MAHRP (PF3D7_1370300)). Pie charts show proportions of each *var* transcript and the size of the chart is proportional to the total RCN. * denotes significant difference of p < 0.01.

*Var* transcription patterns in these three lab strains were compared via quantitative reverse-transcriptase PCR (q-RTPCR) using primers for almost every member of the fully-sequenced *var* gene family of the strains 3D7 and NF54 [12], and of FCR-3. This assessed the specific subsets of *var* genes expressed in each strain. Strikingly, the clones from the different strains expressed very different *var* subsets (Fig. 1A). Statistical analyses demonstrated that *var* subsets expressed by 3D7 were significantly different from both NF54 and FCR-3 (Fig. 1A, S1 Table and S2A-C Tables). 3D7 expressed predominantly upsA and upsB types (B, BC and BA)). In contrast, neither NF54 nor FCR-3 highly expressed upsA transcripts (S2B and S2C Tables).

The difference between the strains was particularly striking considering that 3D7 was originally considered to be a clone derived from the NF54 isolate, although the NF54 isolate has since been reported to contain two closely-related genotypes, 3D7 and ‘E5’ [44]. In fact, we found that in all cases, the total level of *var* transcription was about ten times higher in 3D7 than in NF54 (Fig. 1B). Furthermore, NF54 clones tended to express single dominant *var* transcripts, whereas all 3D7 clones, prepared identically and cultured for a similar number of cycles, expressed several transcripts (S1 Fig.).

### Sirtuin expression is variable between *P*. *falciparum* strains

**Fig 2:**
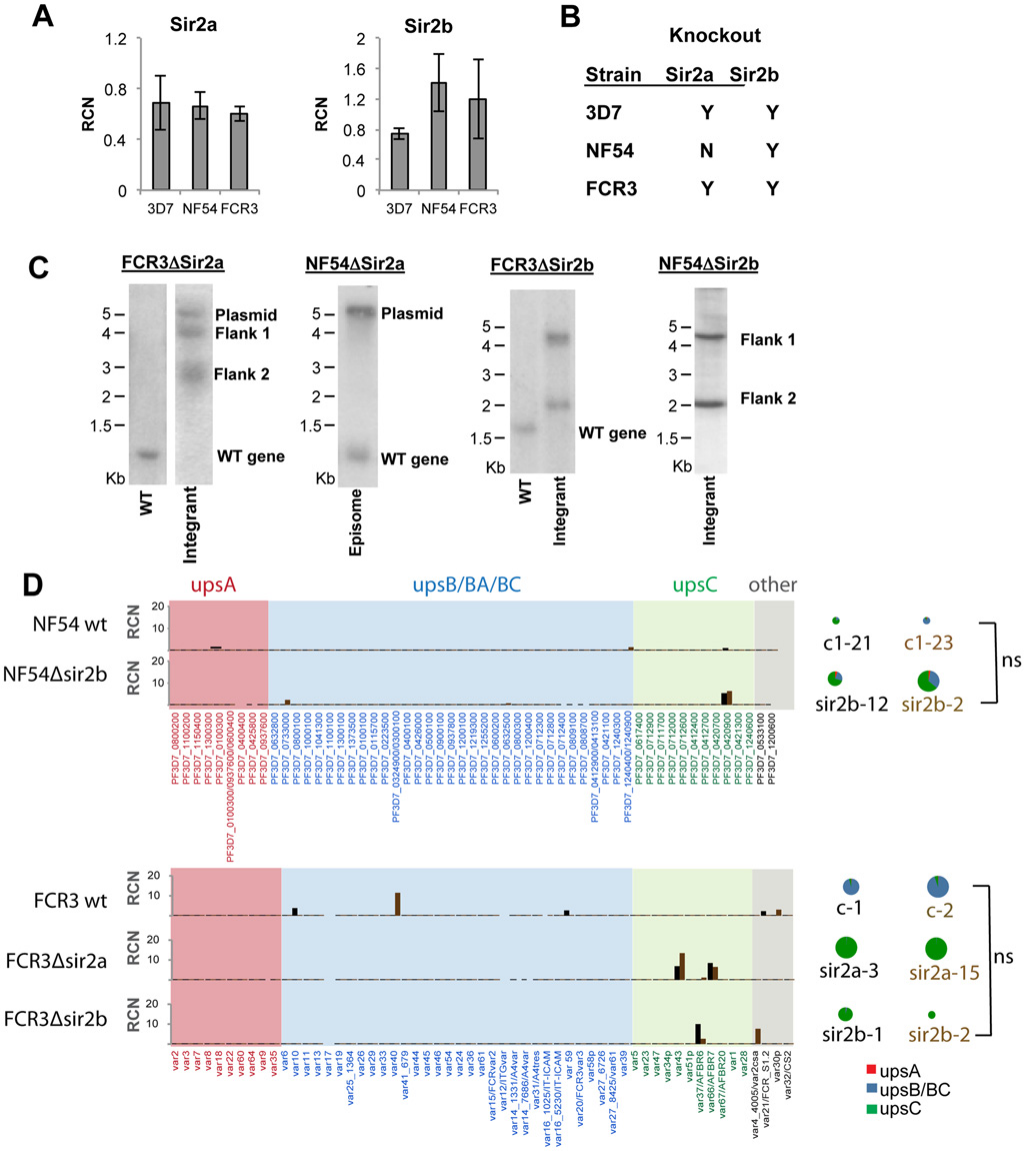
Sirtuin expression and genetic targeting in NF54 and FCR-3. A: Quantitative RT-PCR analysis of *PfSir2a* and *PfSir2b* expression in schizont-stage 3D7, NF54 and FCR-3. RCN is calculated by comparison to the average abundance of transcripts encoding two control genes: seryl-tRNA-synthetase and myosin. Plot shows average of 2 biological replicates, measured in technical duplicate. B: Table showing sirtuin gene disruptions attempted and achieved in 3D7, NF54 and FCR-3. C: Southern blots showing disruption of *PfSir2b* in NF54, *PfSir2a* and *PfSir2b* in FCR-3, and attempted but unsuccessful disruption of *PfSir2a* in NF54. The expected sizes for the FCR-3Δ*sir2a* and NF54Δ*sir2a* endogenous genomic locus, integrated flank 1, integrated flank 2 and concatamerized plasmid following AccI/NdeI digestion are 1.1 kb, 3.9 kb, 2.4 kb and 5.2 kb, respectively. The expected sizes for the FCR-3Δ*sir2b* and NF54Δ*sir2b* endogenous genomic locus, integrated flank 1, integrated flank 2 and concatamerized plasmid following ScaI/HpaII digestion are 1.7 kb, 4.2 kb, 2.1 kb and 4.5 kb. D: *Var* expression patterns in two clones each of NF54 wt, NF54Δ*sir2b*, FCR-3 wt, FCR-3Δ*sir2a* and FCR-3Δ*sir2b* measured by qRT-PCR using gene-specific primers for the majority of the *var* gene family, sorted by ups-group. RCN was calculated by comparison to the abundance of transcripts encoding the average of the three control genes seryl-tRNA synthetase, arginyl-tRNA synthetase and glutaminyl-tRNA synthetase. Individual clones denoted in black and brown bars. Pie charts define percentage of each ups class expressed, and are sized proportional to the total RCN. ns = no significant difference between total *var* gene expression levels in the individual strains.

The only proteins known thus far to have a direct effect on *var* expression patterns are chromatin-modifying proteins, including heterochromatin protein 1 [45]; histone methyltransferases [46] and the histone deacetylases PfHda2 [47], PfSir2a and PfSir2b [4,23,30]. Focusing on the sirtuins, both PfSir2a and PfSir2b are encoded in the genomes of 3D7, NF54 and FCR-3 and their expression was therefore measured to see if variation at the transcriptional level might correlate with the observed differences in *var* expression. All three strains expressed both sirtuins, although the precise balance of expression levels varied, with a trend (albeit non-significant) toward lower expression of *PfSir2b* in 3D7 than in NF54 and FCR-3 (Fig. 2A).

### Disruption of PfSir2a in FCR-3 and disruption of PfSir2b in FCR-3 and NF54 does not alter *var* gene expression as dramatically as in 3D7

To investigate the *var* expression phenotypes caused by sirtuin knockouts in various strain backgrounds, gene disruption by homologous recombination was attempted for both *PfSir2a* and *PfSir2b* in 3D7, FCR-3 and NF54 (Fig. 2B and 2C, S2 Fig.). Both knockouts are previously published for 3D7 [4,23] (as with any gene disruption strategy, a low level of disrupted or unstable transcripts may still be produced in these lines, but Duraisingh *et al*. detected no stable full-length transcript by Northern blot, and previously-published immunofluorescence data has also shown the absence of Sir2a protein in this mutant line [48]). Both *PfSir2a* and *b* disruptions were successfully obtained in FCR-3 but it was only possible to disrupt *PfSir2b* in NF54; drug selection and cycling for over a year failed to disrupt *PfSir2a* in the NF54 strain (Fig. 2C), providing some evidence that it might be essential in this parasite genetic background.

The disruption of *PfSir2a* has previously been shown to cause extensive relaxation of *var* gene silencing in 3D7, with clonal parasites expressing a fixed set of ~10 *var* genes and also having an elevated total transcript level [30]. Here, by contrast, FCR-3Δ*PfSir2a* showed no significant change in overall transcript abundance or in the number of genes expressed in a single clone, albeit both clones switched to an upsC *var* transcript (Fig. 2D and S3A Table).

Previously, changes in *var* gene expression were also observed following disruption of *PfSir2b* in 3D7 [4]. The NF54 and FCR-3 *PfSir2b* knockouts were likewise assessed for *var* expression (Fig. 2D). No gross alterations in the dominant *var* expression patterns were observed (S3A and S3B Tables) although there were some subtle changes: some NF54Δ*PfSir2b* clones expressed more than one gene at detectable levels and there was also a slight increase in the level of total *var* transcripts compared to WT NF54, whereas FCR-3Δ*PfSir2b* showed a slight decrease in total transcripts. All of the *Sir2b* gene-disruption clones in NF54 and FCR-3 switched to predominantly upsC transcripts.

### Telomere maintenance is affected by loss of either sirtuin

**Fig 3:**
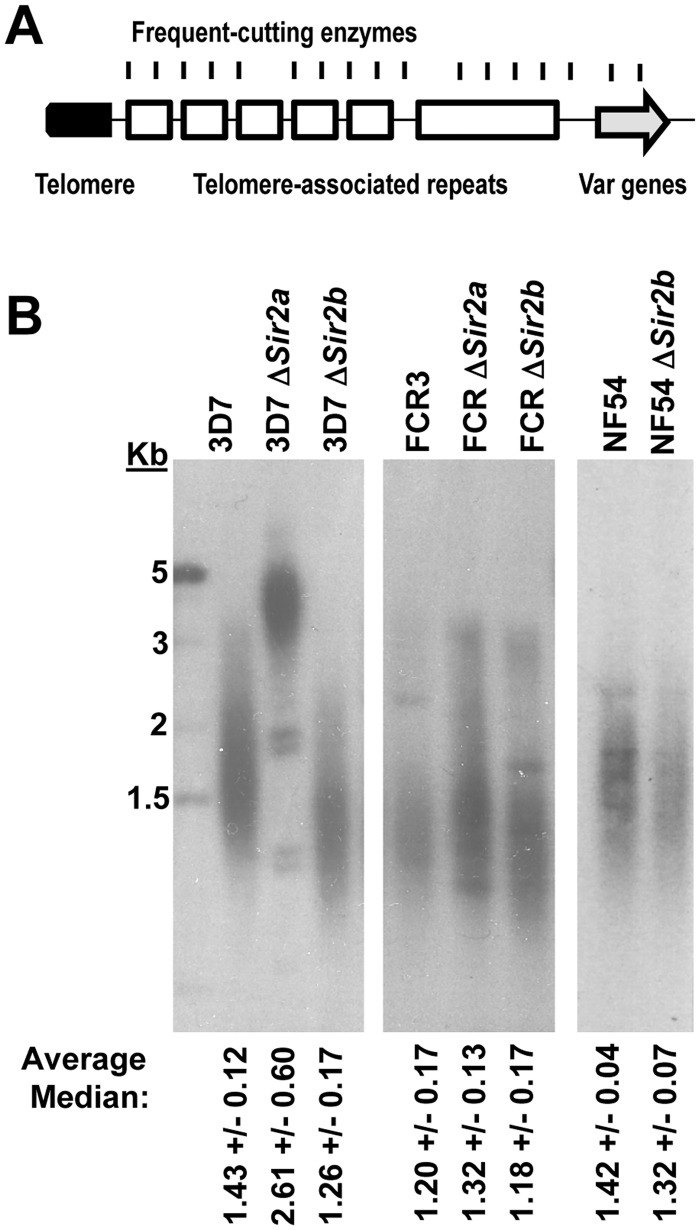
Telomere length is slightly altered in NF54 and FCR-3 sirtuin mutants. A: Schematic showing structure of a *P*. *falciparum* telomere. The genome is digested with frequent-cutting restriction enzymes which do not cut within the telomere repeat to make a Telomere Restriction Fragment (TRF) Southern blot. B: TRF Southern blots of sirtuin-mutant lines compared to parental lines. Representative blots are shown and represent more than 3 experiments for each strain. The average median telomere lengths +/- S.D. from replicate blots are listed below each smear.

Telomere elongation has previously been reported in 3D7Δ*PfSir2a* [4], therefore, telomere lengths were examined in all sirtuin mutant strains using Telomere Restriction Fragment (TRF) Southern blotting (Fig. 3A). Comparing densitometry measurements from at least three replicate blots, 3D7Δ*PfSir2a* did indeed show highly elongated telomeres, as well as several distinct bands that may possibly represent telomeric repeats that have recombined into chromosome-internal locations and are now ‘fixed’, whereas FCR-3Δ*PfSir2a* showed only a slight elongation (Fig. 3B). The phenotypes of elongated telomeres was stable after many months of 3D7 growth in culture (S3A Fig.). In 3D7, loss of *PfSir2b* had the opposite effect to loss of *PfSir2a*, shortening the median telomere length by ~0.2 kb. A marginal telomere decrease (0.1 kb) was observed in NF54Δ*PfSir2b*. Although many of the telomeres in FCR-3Δ*PfSir2b* did clearly decrease in size, the bulk population remained unchanged due to the presence of a small but distinct population of long telomeres (Fig. 3B).

### Complementation of 3D7ΔSir2a with wildtype Sir2a reverses the level of *var* gene expression

**Fig 4:**
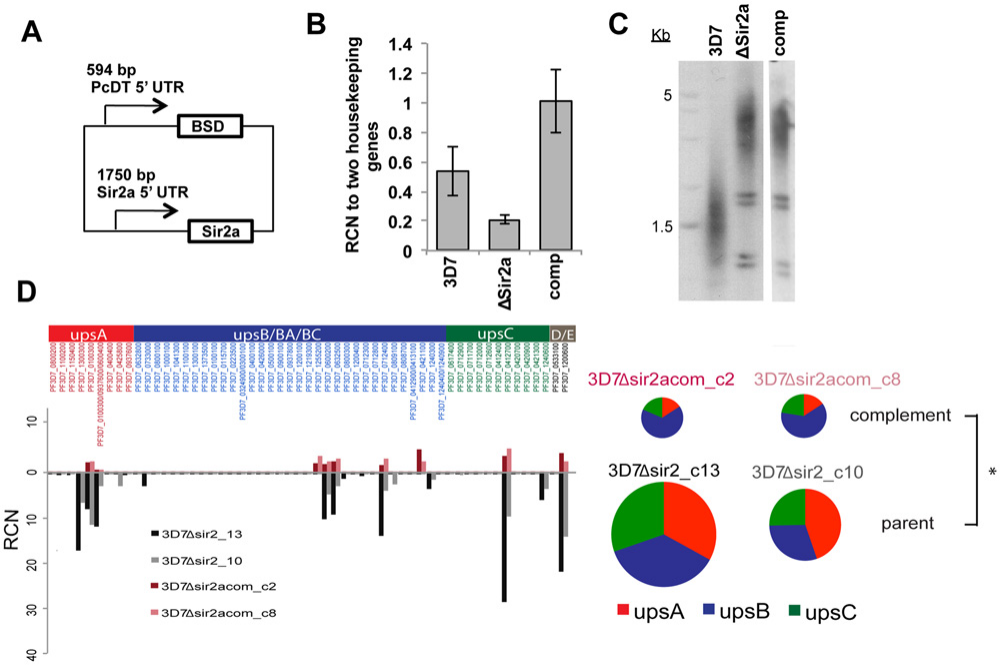
Complementation of *3D7ΔSir2a* results in reduced *var* gene expression. A: Schematic of *PfSir2a* complementation plasmid. B: *PfSir2a* expression levels measured by qRT-PCR in schizonts of 3D7, 3D7Δ*Sir2a* and 3D7Δ*Sir2a*_comp. RCN is calculated by comparison to the average abundance of transcripts encoding two control genes: seryl-tRNA-synthetase and myosin. Plot shows average of 2 biological replicates measured in technical duplicate. C: TRF Southern blot of 3D7, 3D7Δ*Sir2a* and 3D7Δ*Sir2a*_comp. D: *Var* expression patterns in 2 clones each of 3D7Δ*Sir2a* (red, pink) and 3D7Δ*Sir2a*_comp (black, grey), measured as in Fig. 2D. 3D7Δ*Sir2a*_comp shows significant reduction in *var* expression levels as compared to 3D7Δ*Sir2a*. Pie charts represent *var* expression by ups class. * denotes significant difference of p < 0.01.

Since 3D7Δ*PfSir2a* showed the most dramatic phenotypes in both *var* expression and telomere length, complementation of this mutant was attempted. *PfSir2a* was cloned under the endogenous upstream sequence from the *PfSir2a* gene and transfected episomally into 3D7Δ*PfSir2a* (Fig. 4A). *PfSir2a* was expressed at about twice its original level in ‘3D7Δ*PfSir2a* _*comp*’, while only a fragment of the disrupted gene was expressed in the mutant, at about half of wildtype levels (likely due to low-level detection of disrupted transcripts in the mutant) (Fig. 4B & [23]). After more than 30 generations post-transfection, neither telomere elongation nor the deregulated pattern of *var* gene expression was reversed in 3D7Δ*PfSir2a*_*comp* (Fig. 4C and 4D) but the overall level of *var* transcription was significantly lowered (S3C Table). The total *var* transcript levels in the complemented knockout line were comparable to wildtype 3D7.

### Disruption of PfSir2a is associated with extensive genome rearrangement

**Fig 5:**
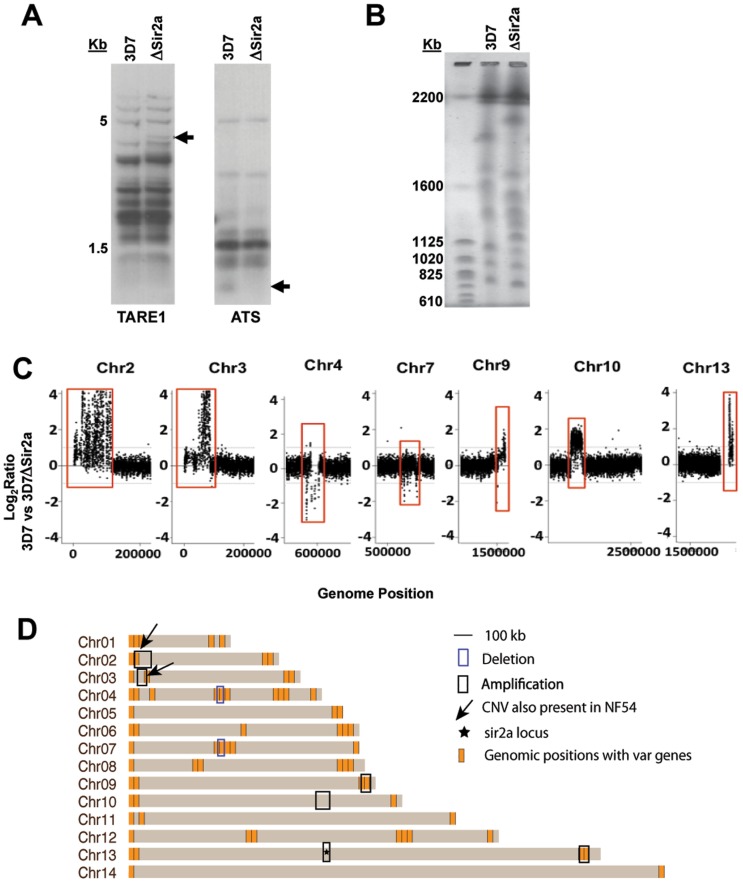
Dramatic chromosomal rearrangements in 3D7Δ*Sir2a* gene disruption mutants. A: Southern blots of genomic DNA from 3D7 and 3D7Δ*Sir2a*, digested with Taq1 and probed for the first sub-telomeric repeat, TARE1, or the conserved ‘ATS’ region of *var* genes. Arrows indicate bands that differ between the two lines. B: Pulsed-field gel electrophoresis showing altered karyotype in 3D7Δ*Sir2a* compared to 3D7. C: Comparative genomic hybridization plots of 3D7 and 3D7Δ*Sir2a*. Only those chromosomes containing significant CNVs are shown. The log_2_ratio of Cy3/Cy5 value is plotted against chromosomal position. D: Chromosomal rearrangements in 3D7Δ*Sir2a* detected by array CGH. The location of each *var* gene is shown in orange in the schematic chromosomes. The two locations that show CNV in NF54 are indicated with arrows. Deletions and amplifications are highlighted with boxes.

To further understand the genetic impact of gene disruption in 3D7Δ*PfSir2a* parasites, we examined chromosomal changes that occurred in the knockout strain. Since PfSir2a binds to sub-telomeric DNA [25], Southern blots were performed using genomic DNA from 3D7 and 3D7Δ*PfSir2a* to establish whether changes in sub-telomeric repeat sequences and/or sub-telomeric genes were observed, These Southern blots produced similar, although not identical, profiles in 3D7 and 3D7Δ*PfSir2a* for both the sub-telomeric repeat TARE1 and the conserved ‘ATS’ region of the *var* gene family (Fig. 5A). Pulsed field gel electrophoresis, however, revealed that the karyotype of 3D7Δ*PfSir2a* was extensively altered, with large changes in chromosome sizes encompassing tens or hundreds of kilobases: too large to be accounted for by ~2kb of extra telomere repeat sequence (Fig. 5B). The 3D7Δ*PfSir2a* line is karyotypically identical in a recent clone and in a bulk population (S3B Fig.) suggesting that these chromosomal changes may have occurred due to a major disruption following gene knockout, and were then fixed into a homogenous population of surviving parasites,

To further analyze the changes causing the altered karyotypes, comparative genomic hybridization (CGH) was carried out to pinpoint genetic differences between 3D7 and 3D7Δ*PfSir2a* that could account for their differing *var* transcription patterns. Although no major rearrangement or amplification of *var*-encoding regions had apparently occurred, CGH revealed widespread change throughout many chromosomes in 3D7Δ*PfSir2a* (Fig. 5C and S4 Table). Amplifications and deletions were observed on seven chromosomes. Interestingly, all of the changes were associated with *var* gene dense regions of the chromosome, except one on chromosome 10 (Fig. 5D and S5 Table). It should be noted that the PfSir2a knockout was created by a single-crossover insertion resulting in CGH detection as a genetic amplification, even though no protein product is produced.

CGH comparison was also carried out between 3D7 and NF54. Several copy number variations (CNVs) were detected, mostly as losses in 3D7 or amplifications in NF54. These were primarily in sub-telomeric regions, including the previously-noted chromosome 2 truncation which encompasses Knob-associated Histidine-Rich Protein and Erythrocyte Membrane Protein 3 and which is well-tolerated in many isolates [49] (S4 Fig. and S6 Table). The changes in chromosome 2 and 3 of both 3D7Δ*PfSir2a* and NF54 likely occur spontaneously, suggesting that these regions are unstable and therefore may not have a connection to the PfSir2a knockout. Additional changes detected in the 3D7 versus 3D7Δ*PfSir2a* analysis were, however, much more extensive than in NF54 versus 3D7, although the mutant strain had been separated from its parent for only a few months, compared to many years for NF54. No clear candidate emerged with a strong potential link to the regulation of *var* transcription but the roles of the genes affected by PfSir2a knockout will be pursued in further work.

## Discussion

The expression and silencing of adhesin-encoding *var* genes is a key virulence factor in *P*. *falciparum* and the sirtuins were until very recently the only proteins reported to directly affect this process. This study evaluated and compared the *var* expression patterns and phenotypes of sirtuin mutants in three laboratory strains. *Var* expression patterns were found to be highly variable, both qualitatively and quantitatively, while the sirtuins had pleiotropic effects upon *var* gene expression, telomere maintenance and genome stability. The sensitivity of different strains to altered sirtuin levels, however, varied significantly with *PfSir2a* knockout in 3D7 having the most severe impact and NF54 and FCR-3 knockouts demonstrating a more minimal effect.

This side-by-side comparison of *var* gene expression in several laboratory strains helps to resolve what has been an ongoing conflict between reports of mutual exclusion and poly-genic *var* expression patterns. Both modes apparently exist in different strains; NF54 and FCR-3 show tighter mutually exclusive expression than 3D7, which transcribes several *var* genes quite abundantly at a similar age post-cloning. The concept that *var* expression is strictly mutually exclusive was initially derived from reverse transcription and cloning of *var* cDNAs from lines of FCR-3 selected for different adhesive phenotypes. This previous analysis detected only a single *var* gene per adhesive phenotype [22] but the proposed model has since been debated. In particular, single-cell RT-PCR experiments detected several *var* transcripts in single cells of FCR-3 [17,50]; while in 3D7, poly-allelic *var* expression has been demonstrated in single cells by quantitative PCR and RNA-FISH at the mRNA level, immunofluorescence at the protein level and cytoadherence assays at the phenotypic level [20]. Clones of 3D7 have, furthermore, been monitored for switching over many cycles, showing that the expression of multiple *var* genes is quite stable and hence not consistent simply with rapid switching [30] (although other models, such as very rapid on/off switching amongst largely the same subset of *var* genes, cannot be entirely excluded without further experimental validation). NF54 however, was independently reported to conform to the mutual exclusion model [21,31,51]. Since 3D7 was originally a clone of NF54, *var* expression patterns must have altered in one or both of these lines after lengthy *in vitro* culture. The original *ex vivo* phenotype is not recorded and it is not clear whether the 3D7 or NF54 type of allelic expression more closely represents that found in *in vivo* infections.

A second novel finding from this work is the dramatic disparity in *var* transcript levels between 3D7 and NF54. Again, it is not known whether either strain represents the ‘real’ level of *var* transcription *in vivo*, although it has been reported that *var* transcription falls rapidly after parasites are transferred to *in vitro* culture, suggesting that the higher level may be closer to reality [52]. In either case, the disparity suggests that a *var*-specific transcription factor or other regulators, such as chromatin proteins, may exist at very different levels in the two cultured strains. CGH revealed no obvious candidate at the genetic level, although there were several CNVs in these two strains. The majority of CNVs represented losses of sub-telomeric genes, which are probably dispensable in *in vitro* culture. The remaining genes require further investigation but exhibit no obvious link to *var* transcription.

Turning to the effects of sirtuins on *var* gene expression and genome stability, these could all stem mechanistically from histone deacetylase activity, although additional enzymatic targets or structural roles for sirtuins remain possible. Sirtuins in model organisms generate sub-telomeric heterochromatin via histone deacetylation, silencing sub-telomeric genes and probably also regulating access for telomerase. Telomere maintenance is, indeed, affected by sirtuin complexes in species from budding yeast to mammals, e.g. telomere shortening is seen after loss of human SIRT1 [27,53]. Thus, the relaxation of *var* gene silencing and the altered telomere lengths observed in sirtuin mutants could both be explained by altered acetylation and relaxation of sub-telomeric heterochromatin. (Telomere length between clones has recently been shown to vary in parasites grown under drug pressure and this may also have an influence on telomere maintenance [54].) Interestingly, truncated *P*. *falciparum* chromosomes which lack their sub-telomeric repeats also show abnormally elongated telomeres, perhaps because sirtuin binding is reduced on these chromosomes [36]. PfSir2a localizes to nucleoli as well as telomeres, where it was recently reported to act as a negative regulator of ribosomal RNA gene transcription in 3D7 [55]. Again, this effect was apparently due to altered histone acetylation upstream of rRNA genes.

The third phenotype of chromosomal rearrangement seen in 3D7Δ*PfSir2a* may result from a crisis in genome stability caused by altered sirtuin activity, although the exact timing of unfolding of this phenotype cannot be determined simply because genetic manipulation in *P*. *falciparum* is a very lengthy and unpredictable process. Again, such a ‘crisis’ could be due to altered histone acetylation, although other roles for PfSir2a in chromatin structure or the regulation of DNA replication remain possible. A human sirtuin, SirT6, targets histones in DNA sequences that are difficult to replicate, including telomeres, and the absence of SirT6 causes stochastic chromosome breaks and fusions [56]. This phenotype is significantly similar, although not identical, to that observed in 3D7Δ*PfSir2a*. If a genome ‘crisis’ did occur when 3D7Δ*PfSir2a* was generated, it must have been resolved, resulting in a stable karyotype that permits *in vitro* survival (and yet that cannot directly account for the dysregulated *var* gene expression phenotype simply in terms of missing or amplified genes). However, the failure to disrupt *PfSir2a* in NF54 suggests that this may be a rare and difficult event. Nevertheless, remarkable levels of genome variation are apparently tolerated in *P*. *falciparum* [57], certain large deletions are commonly observed in *in vitro* culture [49] and it is notable that the karyotypes of different strains, including 3D7 and FCR-3, are not identical ([58,59] and data not shown). Pressures to retain a particular karyotype may be greater *in vivo* than *in vitro*, yet it has been observed that other pathogens such as *Candida glabrata* also have surprisingly flexible genomes even *in vivo*, perhaps as an adaptive strategy [60].

All phenotypes relating to PfSir2a were seen more dramatically in 3D7 than in FCR-3, suggesting that 3D7 is significantly more sensitive to changes in PfSir2a activity. The difference could reflect a spectrum of sirtuin-sensitivity existing in the wild, or changes following lab adaptation. Indeed, a strong effect of genetic background is seen when SIRT1 is mutated in the mouse [28]. One explanation is that PfSir2a and PfSir2b may act more redundantly in FCR-3 than in 3D7; another is that putative PfSir2a-interacting factors may be transcribed at different levels, or may differ in their sirtuin-interacting domains. In *S*. *cerevisiae*, alterations in sirtuin partner proteins can certainly modulate function [61] but no such partners have yet been identified for *P*. *falciparum* sirtuins.

Regarding *PfSir2b*, its mutation caused distinct but related phenotypes to *PfSir2a* mutation and these phenotypes were similar in 3D7, NF54 and FCR-3. Firstly, telomeres were slightly shortened in all three strains, suggesting that the two sirtuins play opposing roles in controlling telomere maintenance. Secondly, in contrast with the dramatic effect on *var* transcription reported in 3D7Δ*PfSir2a*, there was no major change in dominant *var* transcripts, although the silencing of sub-telomeric *var*s was previously reported to be slightly altered in 3D7Δ*PfSir2b* [4], while the clones of FCR-3Δ*PfSir2b* and NF54Δ*PfSir2b* analyzed here had all switched to predominantly upsC *var* genes. PfSir2b remains uncharacterized as an enzyme but its possible roles include deacetylating a distinct set of histone residues to PfSir2a, acting as a structural chromatin element, or perhaps performing both functions in partial overlap with PfSir2a.

Finally, with respect to complementation of 3D7Δ*PfSir2a*, there was a dramatic reversion in the level of *var* gene expression, but mutually exclusive expression of *var* genes was not restored. This effect on *var* gene expression levels was also unlinked from effects on telomere length and genome integrity. The absence of full phenotypic reversion probably results from the extensive genome rearrangements seen in this line. The massive relaxation of *var* gene silencing in 3D7Δ*PfSir2a* may in fact be a complex result of altered TPE and altered histone modification together with altered chromatin context, explaining why sub-telomeric *var* genes are not uniformly turned on and also why the transcriptional pattern is not reversible. Alternatively, the complete ‘re-setting’ of epigenetic control over *var* transcription from a deregulated state may require passage through meiosis. Nevertheless, the striking quantitative reversal of *var* over-expression in the complemented line suggests that PfSir2a has a distinct—and reversible—effect upon the level of *var* transcription. It also argues against the hypothesis that another *var* gene regulator was deleted during chromosome rearrangement and that the phenotypes of the mutant are unrelated to PfSir2a itself, although the nature of this genetic experiment makes it impossible to completely exclude this. It is not clear why the elongated telomeres in 3D7Δ*PfSir2a* could not be re-shortened; however, ‘set-point’ control in telomere maintenance is a subject of ongoing investigation in model systems. For example, aberrantly shortened telomeres in telomerase-mutant human cells cannot be ‘reset’ by simply restoring telomerase [62]. Much remains to be discovered about telomere maintenance in *P*. *falciparum*, particularly since it may form a target for novel anti-malarial drugs.

In conclusion, *var* expression patterns in some of the most commonly-used laboratory strains are shown here to be highly variable, while the sirtuins have pleiotropic—and likewise variable—effects upon *var* expression, telomere maintenance and genome stability. It will now be important to characterize sirtuin-interacting proteins and other chromatin-modifying enzymes in order to better understand the mechanisms underlying antigenic variation in *in vivo* infections with this important human pathogen.

## Supporting Information

S1 Fig Heat map demonstrating individual *var* gene expression within the different clones of 3D7, NF54 and FCR-3. *Var* gene expression was assessed using a 3D7-specific primer subset [12] for 3D7 and NF54 or an FCR-3-specific subset for the *var* family in FCR-3 [63], which contains similar, but not identical, numbers of upsA, B and C type *var* genes.
(PDF)

S2 Fig Generation of PfSir2a and b disruption parasites. A: Strategy for single crossover disruption of *PfSir2a* locus adapted from Duraisingh et al., 2005. Probes used for Southern blot confirmation of integration are shown in red. Dashed lines represent plasmid sequence. A = AccI sites, N = NdeI sites. B: Strategy for single crossover disruption of *PfSir2b* locus adapted from Tonkin et al., 2009. Probes used for Southern blot confirmation of integration are shown in red. Dashed lines represent plasmid sequence. S = ScaI sites, H = HpaII sites.
(PDF)

S3 Fig Comparison of bulk and cloned parasite populations. A: TRF blots showing telomere lengths for parasites in the bulk population versus a recent clone for 3D7 WT and 3D7Δ*sir2a*. B: PFGE showing karyotypes of the bulk population versus a recent clone for 3D7 WT and 3D7Δ*sir2a* (markers = *S*. *cerevisiae* chromosomes).
(PDF)

S4 Fig Comparative genomic hybridization plots of 3D7 and NF54. Only those chromosomes containing significant CNVs are shown. The log_2_ratio of Cy3/Cy5 value is plotted against chromosomal position.
(PDF)

S1 Table RCN values for comparison of *var* gene expression levels between 3D7, NF54 and FCR-3 corresponding to Fig. 1. A: RCN values for *var* gene expression levels in 3D7. B: RCN values for *var* gene expression levels in NF54. C: RCN values for *var* gene expression levels in FCR-3.
(XLSX)

S2 Table Statistical test results of the differences in *var* gene expression levels between 3D7, NF54 and FCR-3. A: Results of non-parametric tests (unpaired Wilcoxon test) performed between the total *var* gene expression levels of 3D7 and NF54. B: Results of pairwise Chi-squared tests performed between the averaged expression levels of (upsA, upsB and upsC) between 3D7, NF54 and FCR-3. C: Results of pairwise Chi-squared tests of the differences in *var* gene expression patterns (composition of upsA, upsB and upsC) between clones of 3 strains.
(PDF)

S3 Table Statistical test results of var gene expression levels between various strains. A: Paired Wilcoxon tests were performed between the individual *var* gene expression levels of different FCR-3, FCR-3Δ*sir2a* and FCR-3Δ*sir2b* strains. No significant *var* gene expression differences were detected. B: Paired Wilcoxon tests were performed between the individual *var* gene expression levels of NF54 and NF54Δ*sir2b* strains. No significant *var* gene expression differences were detected. C: Paired Wilcoxon tests were performed between the individual *var* gene expression levels of different strains. Significant reduction in *var* gene expression occurs between 3D7Δ*sir2a*-comp and 3D7Δ*sir2a*.
(PDF)

S4 Table CNVs detected in 3D7Δ*sir2a* (vs 3D7): Comparative Genomic Hybridization. (PDF)

S5 Table *P. falciparum* genes in the genomic region that showed copy number change in 3D7Δ*sir2a* (vs 3D7) on chromosome 10: Comparative Genomic Hybridization. (PDF)

S6 Table CNVs in NF54 (vs 3D7): Comparative Genomic Hybridization. (PDF)

S7 Table Primers used in this study. (PDF)

S8 Table *var* gene nomenclature and correction factors for qRT-PCR primer efficiency. (PDF)
