# Supplementary figures and images for: Functional Analysis of Sirtuin Genes in Multiple Plasmodium falciparum Strains
Source: PLoS One. 2015 Mar 17;10(3):e0118865. doi: 10.1371/journal.pone.0118865 (PMC4364008; doi:10.1371/journal.pone.0118865)

S1 Fig. Merrick et al.

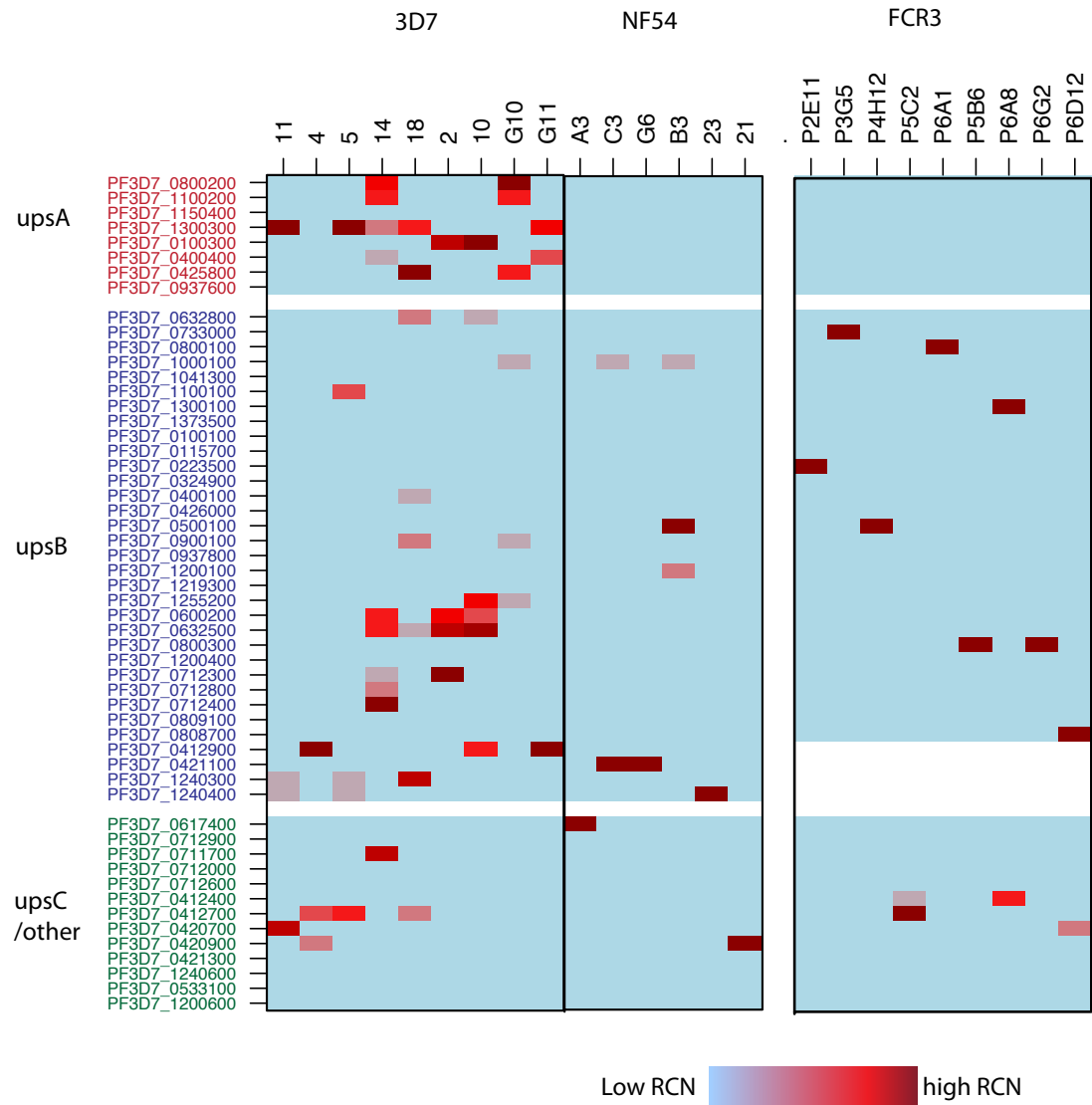

Supplement: S1 Fig — Var gene expression was assessed using a 3D7-specific primer subset [12] for 3D7 and NF54 or an FCR-3-specific subset for the var family in FCR-3 [63], which contains similar, but not identical, numbers of upsA, B and C type var genes. (PDF) [file pone.0118865.s001.pdf]

S2 Fig. Merrick et al.

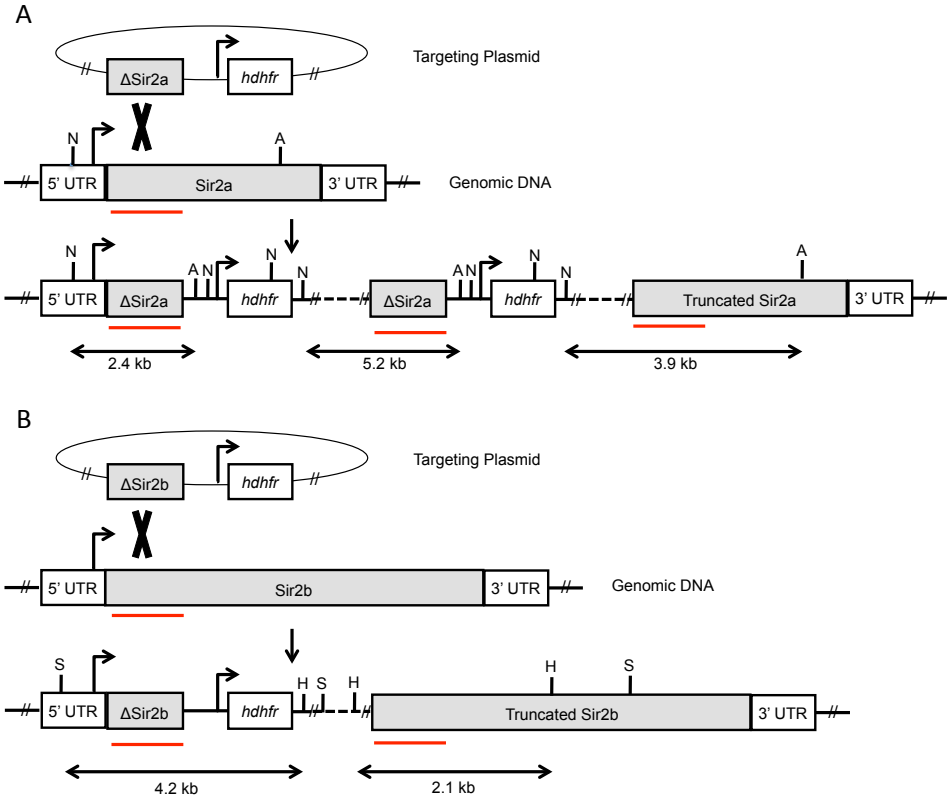

Supplement: S2 Fig — A: Strategy for single crossover disruption of PfSir2a locus adapted from Duraisingh et al., 2005. Probes used for Southern blot confirmation of integration are shown in red. Dashed lines represent plasmid sequence. A = AccI sites, N = NdeI sites. B: Strategy for single crossover disruption of PfSir2b locus adapted from Tonkin et al., 2009. Probes used for Southern blot confirmation of integration are shown in red. Dashed lines represent plasmid sequence. S = ScaI sites, H = HpaII sites. (PDF) [file pone.0118865.s002.pdf]

S3 Fig. Merrick et al.

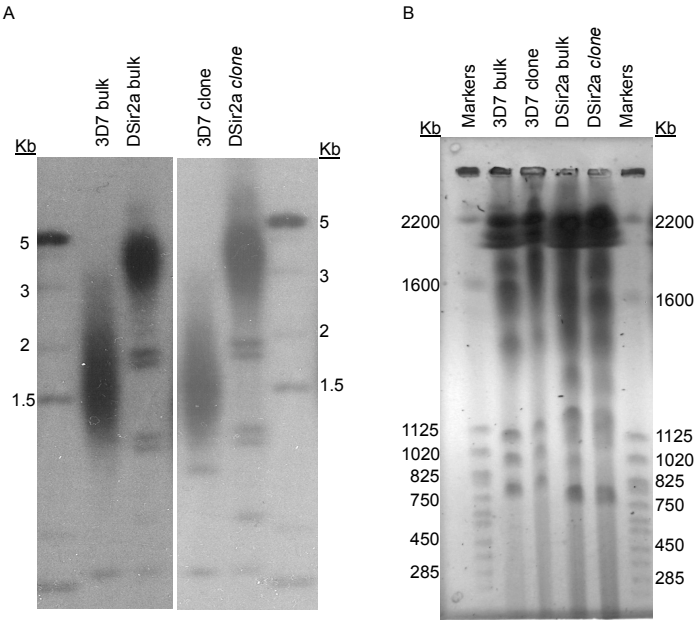

Supplement: S3 Fig — A: TRF blots showing telomere lengths for parasites in the bulk population versus a recent clone for 3D7 WT and 3D7Δsir2a. B: PFGE showing karyotypes of the bulk population versus a recent clone for 3D7 WT and 3D7Δsir2a (markers = S. cerevisiae chromosomes). (PDF) [file pone.0118865.s003.pdf]

S4 Fig. Merrick et al.

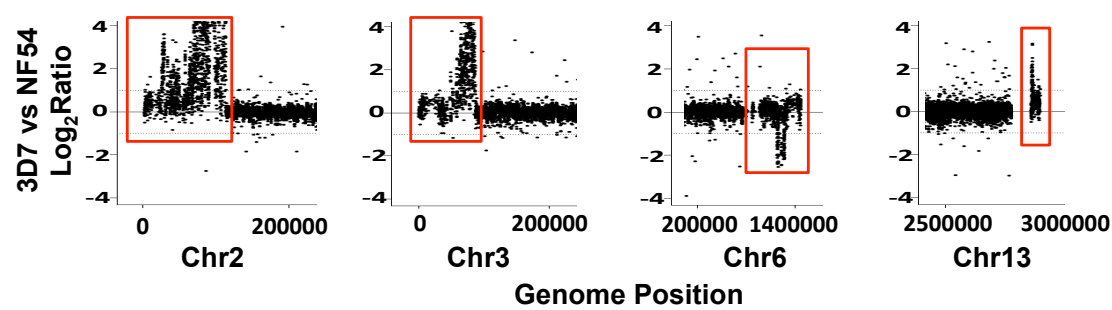

Supplement: S4 Fig — Only those chromosomes containing significant CNVs are shown. The log2ratio of Cy3/Cy5 value is plotted against chromosomal position. (PDF) [file pone.0118865.s004.pdf]
