# Supplementary material for: Functional Analysis of Sirtuin Genes in Multiple Plasmodium falciparum Strains
Source: PLoS One. 2015 Mar 17;10(3):e0118865. doi: 10.1371/journal.pone.0118865 (PMC4364008; doi:10.1371/journal.pone.0118865)
Supplement: S2 Table — A: Results of non-parametric tests (unpaired Wilcoxon test) performed between the total var gene expression levels of 3D7 and NF54. B: Results of pairwise Chi-squared tests performed between the averaged expression levels of (upsA, upsB and upsC) between 3D7, NF54 and FCR-3. C: Results of pairwise Chi-squared tests of the differences in var gene expression patterns (composition of upsA, upsB and upsC) between clones of 3 strains. (PDF) [file pone.0118865.s006.pdf]

S2 Table - Statistical test results of the differences in *var* gene expression levels between 3D7, NF54 and FCR-3

A. Results of non-parametric tests (unpaired Wilcoxon test) performed between the total *var* gene expression levels of 3D7 and NF54.

Strains[1]	3D7	NF54
3D7	1.000	
NF54	0.002[*]	1.000

[*] Significant ($p < 0.01$) differences are shaded gray.

B. Results of pairwise Chi-squared tests performed between the averaged expression levels of (upsA, upsB and upsC) between 3D7, NF54 and FCR-3.

Strains[1]	3D7	NF54	FCR3
3D7	1.000		
NF54	<0.001[*]	1.000	
FCR3	<0.001[*]	0.0012	1.000

[*] Significant ($p < 0.01$) differences are shaded gray.

C. Results of pairwise Chi-squared tests of the differences in *var* gene expression patterns (composition of upsA, upsB and upsC) between clones of 3 strains.

strain A vs strain B	3D7_11	3D7_4	3D7_5	3D7_14	3D7_18	3D7_2	3D7_10	3D7_G10	3D7_G11	NF54_A3	NF54_C3	NF54_G6	NF54_B3	NF54_23	NF54_21	P2E11	P3G5	P4H12	P5C2	P6A1	P5B6	P6A8	P6G2	P6D12
3D7_11	1.00																							
3D7_4	0.08	1.00																						
3D7_5	0.14	0.04	1.00																					
3D7_14	0.79	0.12	0.72	1.00																				
3D7_18	0.00	0.00	0.37	0.28	1.00																			
3D7_2	0.02	0.00	0.04	0.12	0.07	1.00																		
3D7_10	0.02	0.02	0.28	0.17	0.59	0.21	1.00																	
3D7_G10	0.00	0.00	0.10	0.07	0.42	0.31	0.84	1.00																
3D7_G11	0.71	0.20	0.80	0.86	0.83	0.81	0.64	0.78	1.00															
NF54_A3	0.59	0.79	0.35	0.47	0.12	0.01	0.03	0.01	0.39	1.00														
NF54_C3	0.54	0.83	0.86	0.70	0.89	0.54	0.79	0.81	0.76	0.68	1.00													
NF54_G6	0.22	0.73	0.64	0.45	0.51	0.06	0.27	0.16	0.51	0.63	0.98	1.00												
NF54_B3	0.01	0.17	0.15	0.08	0.24	0.03	0.39	0.22	0.30	0.33	0.97	0.71	1.00											
NF54_23	0.48	0.83	0.83	0.66	0.83	0.36	0.57	0.61	0.70	0.68	0.99	0.99	0.91	1.00										
NF54_21	0.46	0.79	0.23	0.35	0.04	0.00	0.02	0.00	0.32	0.96	0.71	0.60	0.30	0.70	1.00									
P2E11	0.00	0.01	0.00	0.00	0.00	0.00	0.03	0.00	0.02	0.11	0.73	0.26	0.79	0.62	0.08	1.00								
P3G5	0.00	0.00	0.00	0.00	0.00	0.00	0.00	0.00	0.00	0.01	0.52	0.05	0.55	0.34	0.00	0.86	1.00							
P4H12	0.00	0.00	0.00	0.00	0.00	0.00	0.02	0.00	0.01	0.02	0.48	0.09	0.57	0.31	0.02	0.80	0.97	1.00						
P5C2	0.02	0.27	0.00	0.01	0.00	0.00	0.00	0.00	0.02	0.96	0.50	0.21	0.06	0.45	1.00	0.00	0.00	0.00	1.00					
P6A1	0.00	0.00	0.00	0.00	0.00	0.00	0.00	0.00	0.00	0.03	0.80	0.13	0.81	0.65	0.01	0.97	0.77	0.76	0.00	1.00				
P5B6	0.00	0.06	0.04	0.03	0.07	0.00	0.17	0.06	0.15	0.28	0.94	0.60	0.97	0.89	0.23	0.87	0.56	0.57	0.03	0.86	1.00			
P6A8	0.00	0.00	0.00	0.00	0.00	0.00	0.00	0.00	0.00	0.50	0.48	0.11	0.38	0.41	0.38	0.34	0.06	0.15	0.01	0.04	0.49	1.00		
P6G2	0.00	0.00	0.00	0.00	0.00	0.00	0.00	0.00	0.00	0.01	0.35	0.02	0.41	0.23	0.00	0.87	0.97	0.94	0.00	0.72	0.49	0.03	1.00	
P6D12	0.00	0.00	0.00	0.00	0.00	0.00	0.00	0.00	0.00	0.32	0.11	0.03	0.21	0.09	0.18	0.53	0.17	0.31	0.00	0.15	0.39	0.62	0.12	1.00

[+] Significant ($p < 0.01$) differences are shaded gray.
