# Supplementary material for: Functional Analysis of Sirtuin Genes in Multiple Plasmodium falciparum Strains
Source: PLoS One. 2015 Mar 17;10(3):e0118865. doi: 10.1371/journal.pone.0118865 (PMC4364008; doi:10.1371/journal.pone.0118865)
Supplement: S3 Table — A: Paired Wilcoxon tests were performed between the individual var gene expression levels of different FCR-3, FCR-3Δsir2a and FCR-3Δsir2b strains. No significant var gene expression differences were detected. B: Paired Wilcoxon tests were performed between the individual var gene expression levels of NF54 and NF54Δsir2b strains. No significant var gene expression differences were detected. C: Paired Wilcoxon tests were performed between the individual var gene expression levels of different strains. Significant reduction in var gene expression occurs between 3D7Δsir2a-comp and 3D7Δsir2a. (PDF) [file pone.0118865.s007.pdf]

S3 Table – Statistical test results of *var* gene expression levels between individual strains

A. Paired Wilcoxon tests were performed between the individual strain *var* gene expression levels of different FCR-3, FCR-3sir2a and FCR-3sir2b strains. No significant *var* gene expression differences between the strains were detected.

Strains+	FCR3.Cl.1	FCR3.Cl.2	FCR3Sir2aX	FCR3Sir2aY	FCR3Sir2b.Cl.1	FCR3Sir2b.Cl.2
FCR3.Cl.1	-					
FCR3.Cl.2	1.00	-				
FCR3Sir2a3	0.81	1.00	-			
FCR3Sir2a15	0.84	0.81	0.75	-		
FCR3Sir2b.Cl.1	0.88	0.75	1.00	0.75	-	
FCR3Sir2b.Cl.2	1.00	0.88	0.88	0.88	1.00	-

B. Paired Wilcoxon tests were performed between the individual strain *var* gene expression levels of NF54 and NF54 sir2b strains. No significant *var* gene expression differences between strains were detected.

Strains+	NF54.Cl.21	NF54.Cl.23.cf.5	NF54.sir2b12	NF54.sir2b2
NF54.Cl.21	-			
NF54.Cl.23	1	-		
NF54.sir2b12	0.5	0.75	-	
NF54.sir2b2	1	1	0.5	-

C. Paired Wilcoxon tests were performed between the individual *var* gene expression levels of different strains. Significant reduction in *var* gene expression occurs between 3D7sir2acom and 3D7sir2a.

Strains+	3d7sir2acom_c2	3d7sir2acom_c8	3d7sir2_13	3d7sir2_10
3d7sir2acom_c2	-			
3d7sir2acom_c8	0.742	-		
3d7sir2_13	0.008*	0.007*	-	
3d7sir2_10	0.008*	0.008*	0.773	-

* Significant ($p < 0.01$) differences are shaded gray.
