# Supplementary material for: Functional Analysis of Sirtuin Genes in Multiple Plasmodium falciparum Strains
Source: PLoS One. 2015 Mar 17;10(3):e0118865. doi: 10.1371/journal.pone.0118865 (PMC4364008; doi:10.1371/journal.pone.0118865)
Supplement: S4 Table — (PDF) [file pone.0118865.s008.pdf]

# S4 Table – CNVs detected in 3D7deltaSir2a (vs 3D7): Comparative Genomic Hybridization

| Chromosome | Event | | Event size (bps) | Number of probes in event | Average Log2 Ratio of event | Loss / Gain ^ | Subtelomeric # Non-subtelomeric | Gene ID | Gene product categories |
|---|---|---|---|---|---|---|---|---|---|
| | Start | End | | | | | | | |
| 2 | 1,757 | 25,249 | 23,492 | 179 | 0.79 | Gain | Telomeric | PF3D7_0200100 | erythrocyte membrane protein 1 (PfEMP1) |
| 2 | 25,317 | 28,897 | 3,580 | 75 | 3.53 | Gain | Subtelomeric | PF3D7_0200100 | erythrocyte membrane protein 1 (PfEMP1) |
| 2 | 28,949 | 69,005 | 40,056 | 541 | 1.28 | Gain | Subtelomeric | PF3D7_0200200 - PF3D7_0201300 | erythrocyte membrane protein 1 (PfEMP1), rifin, erythrocyte membrane protein 1 (PfEMP1) - exon2, stevor, erythrocyte membrane protein 1 (PfEMP1) (truncated), stevor(pseudogene) |
| 2 | 69,077 | 90,945 | 21,868 | 405 | 2.77 | Gain | Subtelomeric | PF3D7_0201400 - PF3D7_0201800 | Plasmodium exported protein (hyp10), Plasmodium exported protein (hyp9), Plasmodium exported protein (PHISTb), DnaJ protein (putative), RESA- |
| 2 | 90,993 | 98,489 | 7,496 | 86 | 5.53 | Gain | Subtelomeric | PF3D7_0201900 | erythrocyte membrane protein 3 |
| 2 | 98,653 | 103,317 | 4,664 | 76 | 1.58 | Gain | Subtelomeric | PF3D7_0201900 | erythrocyte membrane protein 3 |
| 2 | 103,353 | 104,921 | 1,568 | 32 | 6.14 | Gain | Subtelomeric | PF3D7_0202000 | knob-associated histidine-rich protein |
| 2 | 104,993 | 113,241 | 8,248 | 110 | 2.79 | Gain | Subtelomeric | PF3D7_0202100 - PF3D7_0202200 | Plasmodium exported protein (PHISTc), Plasmodium exported |
| 3 | 58,077 | 86,653 | 28,576 | 455 | 2.63 | Gain | Subtelomeric | PF3D7_0300600 - PF3D7_0301400 | rifin,serine/threonine protein kinase, FIKK family (FIKK3), acyl-CoA synthetase (ACS2),Plasmodium |
| 4 | 558,277 | 559,441 | 1,164 | 25 | -1.54 | Loss | Subtelomeric | PF3D7_0412600 | rifin, pseudogene |
| 7 | 576,853 | 577,797 | 944 | 16 | -1.01 | Loss | Central core | PF3D7_0712600* | erythrocyte membrane protein 1 (PfEMP1) |
| 9 | 1,505,793 | 1,540,173 | 34,380 | 184 | 0.73 | Gain | Telomeric | PF3D7_0937800* | erythrocyte membrane protein 1 (PfEMP1) |
| **10** | **1,178,673** | **1,241,469** | **62,796** | **1062** | **1.30** | **Gain** | **Central core** | **PF3D7_1028700 - PF3D7_1030300** | **merozoite TRAP-like protein, conserved Plasmodium protein, adenosine deaminase (putative), RAP protein (putative), transcription factor (putative), RNA** |
| 13 | 1,215,131 | 1,215,552 | 421 | 11 | 1.26 | Gain | Central core | PF3D7_1328800 | transcriptional regulatory protein sir2a |
| 13 | 2,859,229 | 2,862,105 | 2,876 | 53 | 2.76 | Gain | Subtelomeric | PF3D7_1372700 | rifin |
| 13 | 2,862,245 | 2,864,445 | 2,200 | 51 | 1.75 | Gain | Subtelomeric | PF3D7_1372800 | stevor |
| 13 | 2,864,529 | 2,894,569 | 30,040 | 192 | 0.86 | Gain | Subtelomeric / Telomeric | PF3D7_1372800 - PF3D7_1373500 | erythrocyte membrane protein 1 (PfEMP1), rifin, stevor, unknown exported protein |

^Shaded cells indicate that the genomic change is shared with NF54 vs 3D7 comparison
#Telomeric subtelomeric regions ae demarcated as given in Mok et al. 2008
*Genes located closest to the region
