# Supplementary material for: Functional Analysis of Sirtuin Genes in Multiple Plasmodium falciparum Strains
Source: PLoS One. 2015 Mar 17;10(3):e0118865. doi: 10.1371/journal.pone.0118865 (PMC4364008; doi:10.1371/journal.pone.0118865)
Supplement: S5 Table — (PDF) [file pone.0118865.s009.pdf]

S5 Table – P. falciparum genes in the genomic region that showed copy number change in 3D7deltaSir2a (versus 3D7) on chromosome 10: Comparative Hybridization

Gene ID	Genomic Location(s)	Organism	Previous ID(s)	Product Description
PF3D7_1028700	Pf3D7_10_v3: 1,181,005 - 1,182,501 (+)	P. falciparum 3D7	PF10_0281	merozoite TRAP-like protein (MTRAP)
PF3D7_1028800	Pf3D7_10_v3: 1,183,487 - 1,183,995 (-)	P. falciparum 3D7	PF10_0282	conserved Plasmodium protein, unknown function
PF3D7_1028900	Pf3D7_10_v3: 1,184,728 - 1,185,946 (-)	P. falciparum 3D7	PF10_0283	conserved Plasmodium protein, unknown function
PF3D7_1029000	Pf3D7_10_v3: 1,186,855 - 1,189,548 (-)	P. falciparum 3D7	PF10_0283a	conserved Plasmodium protein, unknown function, pseudogene
PF3D7_1029100.1	Pf3D7_10_v3: 1,190,426 - 1,195,016 (+)	P. falciparum 3D7	PF10_0284	conserved Plasmodium protein, unknown function
PF3D7_1029100.2	Pf3D7_10_v3: 1,190,426 - 1,195,016 (+)	P. falciparum 3D7	null	conserved Plasmodium protein, unknown function
PF3D7_1029200	Pf3D7_10_v3: 1,195,677 - 1,197,344 (+)	P. falciparum 3D7	PF10_0285	conserved Plasmodium protein, unknown function
PF3D7_1029300	Pf3D7_10_v3: 1,197,825 - 1,200,032 (-)	P. falciparum 3D7	PF10_0286	conserved Plasmodium protein, unknown function
PF3D7_1029400	Pf3D7_10_v3: 1,200,894 - 1,202,399 (-)	P. falciparum 3D7	PF10_0287	conserved Plasmodium protein, unknown function
PF3D7_1029500	Pf3D7_10_v3: 1,204,346 - 1,204,762 (-)	P. falciparum 3D7	PF10_0288	conserved Plasmodium protein, unknown function
PF3D7_1029600	Pf3D7_10_v3: 1,206,302 - 1,207,405 (+)	P. falciparum 3D7	PF10_0289	adenosine deaminase, putative
PF3D7_1029700	Pf3D7_10_v3: 1,208,198 - 1,210,914 (-)	P. falciparum 3D7	PF10_0290	conserved Plasmodium protein, unknown function
PF3D7_1029800	Pf3D7_10_v3: 1,211,658 - 1,212,971 (-)	P. falciparum 3D7	PF10_0291	RAP protein, putative
PF3D7_1029900	Pf3D7_10_v3: 1,217,348 - 1,222,441 (+)	P. falciparum 3D7	PF10_0292	conserved Plasmodium protein, unknown function
PF3D7_1030000	Pf3D7_10_v3: 1,223,375 - 1,224,167 (+)	P. falciparum 3D7	PF10_0293	transcription factor, putative
PF3D7_1030100	Pf3D7_10_v3: 1,224,519 - 1,229,092 (-)	P. falciparum 3D7	PF10_0294	pre-mRNA-splicing factor ATP-dependent RNA helicase PRP22, putative (PRP22)
PF3D7_1030200	Pf3D7_10_v3: 1,230,561 - 1,231,913 (-)	P. falciparum 3D7	PF10_0295	conserved Plasmodium protein, unknown function
PF3D7_1030300	Pf3D7_10_v3: 1,233,667 - 1,237,002 (-)	P. falciparum 3D7	PF10_0296	conserved Plasmodium protein, unknown function
