# Supplementary material for: Functional Analysis of Sirtuin Genes in Multiple Plasmodium falciparum Strains
Source: PLoS One. 2015 Mar 17;10(3):e0118865. doi: 10.1371/journal.pone.0118865 (PMC4364008; doi:10.1371/journal.pone.0118865)
Supplement: S6 Table — (PDF) [file pone.0118865.s010.pdf]

S6 Table – CNVs in NF54 (versus 3D7): Comparative Genomic Hybridization

Chromosome	Event		Event	Number of	Average Log2Ratio	Loss/Gain	Subtelomeric/	Gene ID	Gene product categories
	Start	End	size (bps)	probes in event	of event		Non-subtelomeric		
2	1,757	69,077	67,320	796	0.86	Gain	Telomeric/Telomeric	PF3D7_0200100 - PF3D7_0201400	erythrocyte membrane protein 1, PfEMP1, rifin, erythrocyte membrane protein1 (PfEMP1) - exon2, stevor, erythrocyte membrane protein 1 (PfEMP1) - truncated, stevor - pseudogene, Plasmodium exported protein (hyp10)
2	69,081	90,945	21,864	404	2.57	Gain	Subtelomeric	PF3D7_0201400 - PF3D7_0201800	Plasmodium exported protein (hyp10), Plasmodium exported protein (hyp9), Plasmodium exported protein (PHISTb), DnaJ protein (putative), RESA-like protein with PHIST and DnaJ domains
2	90,993	98,489	7,496	86	4.56	Gain	Subtelomeric	PF3D7_0201900	erythrocyte membrane protein 3
2	98,653	113,237	14,584	217	2.61	Gain	Subtelomeric	PF3D7_0201900 - PF3D7_0202200	erythrocyte membrane protein 3, knob-associated histidine-rich protein, Plasmodium exported protein (PHISTc), Plasmodium exported protein,
3	58,777	70,557	11,780	171	1.70	Gain	Subtelomeric	PF3D7_0300700 - PF3D7_0301000	rifin, acyl-CoA synthetase
3	70,561	86,529	15,968	268	2.67	Gain	Subtelomeric	PF3D7_0301100 - PF3D7_0301500	Plasmodium exported protein (hyp13), serine/threonine protein kinase (FIKK family), alpha/beta hydrolase, Plasmodium exported protein
6	1,363,702	1,366,445	2,743	58	-1.13	Loss	Subtelomeric	PF3D7_0632500	erythrocyte membrane protein 1 (PfEMP1)
6	1,372,280	1,372,657	377	11	-1.81	Loss	Subtelomeric	PF3D7_0632700	rifin
6	1,376,435	1,378,888	2,453	39	-1.37	Loss	Subtelomeric	PF3D7_0632800	erythrocyte membrane protein 1 (PfEMP1)
13	2,859,445	2,862,025	2,580	49	1.37	Gain	Subtelomeric	PF3D7_1373500	erythrocyte membrane protein 1 (PfEMP1)
