# Supplementary material for: Functional Analysis of Sirtuin Genes in Multiple Plasmodium falciparum Strains
Source: PLoS One. 2015 Mar 17;10(3):e0118865. doi: 10.1371/journal.pone.0118865 (PMC4364008; doi:10.1371/journal.pone.0118865)
Supplement: S7 Table — (PDF) [file pone.0118865.s011.pdf]

S7 Table – Primers used in this study

Primers were obtained from Integrated DNA Technologies

**Primer name**	**Forward primer**	**Reverse primer**
**Cloning**		
Sir2a	gatc*cctagg*atgggtaatttaatgatt	gatc*cttaag*ctacattattttcttattttt
Sir2pro	at*gggccc*acttaataacgatag	gatc*cctagg*aatatataattattttaatcttaac
**Quantitative PCR**		
Myosin	ggttcagaggatgggcaacat	agacctgcgccagaaaacta
Sir2a	gggaatgtatttgaagcagt	cgatgtgccaattactaaaa
Sir2b	gtcccggctagctcttatcc	Aattgggcacctaacgattg
