# Supplementary material for: Functional Analysis of Sirtuin Genes in Multiple Plasmodium falciparum Strains
Source: PLoS One. 2015 Mar 17;10(3):e0118865. doi: 10.1371/journal.pone.0118865 (PMC4364008; doi:10.1371/journal.pone.0118865)
Supplement: S8 Table — (PDF) [file pone.0118865.s012.pdf]

S8 Table – *var* gene nomenclature and correction factors for qRT-PCR primer efficiency

Old *var* gene nomenclature	New *var* gene nomenclature	Correction Factor 3D7	Correction Factor NF54
PF08_0141	PF3D7_0800200	-0.09965	0.499041
MAL6P1.1	PF3D7_0632800	0.2707	0.23467
MAL6P1.252	PF3D7_0617400	0.06505	0.142741
MAL6P1.316	PF3D7_0600200	-0.01155	0.147893
MAL6P1.4	PF3D7_0632500	-0.05185	-0.172
MAL7P1.50	PF3D7_0712300	-0.37325	-0.09057
MAL7P1.55	PF3D7_0712800	0.14465	0.325936
MAL7P1.56	PF3D7_0712900	-0.06595	0.243994
PF07_0048	PF3D7_0711700	-0.147	-0.30061
PF07_0049	PF3D7_0712000	-0.04395	0.110853
PF07_0050	PF3D7_0712400	0.0712	0.226181
PF07_0051	PF3D7_0712600	-0.0405	0.15666
PF07_0139	PF3D7_0733000	0.1896	0.576498
PF08_0103	PF3D7_0809100	0.2359	0.295264
PF08_0106	PF3D7_0808700	0.0387	0.794433
PF08_0140	PF3D7_0800300	0.2228	0.595811
PF08_0142	PF3D7_0800100	-0.0244	0.36938
PF10_0001	PF3D7_1000100	-0.0519	0.595811
PF10_0406	PF3D7_1041300	-0.06255	0.134763
PF11_0007	PF3D7_1100100	-0.12295	-0.07899
PF11_0008	PF3D7_1100200	-0.21695	-0.03289
PF11_0521	PF3D7_1150400	-0.2347	-0.67903
PF13_0001	PF3D7_1300100	-0.0573	0.171632
PF13_0003	PF3D7_1300300	-0.87165	-0.85301
PF13_0364	PF3D7_1373500	-2.0081	1.236447
PFA0005w	PF3D7_0100100	-0.67775	-0.40355
PFA0015c	PF3D7_0100300	-1.4292	-0.4266
PFA0015c/PFI1820w/Mal6P1.314	PF3D7_0100300/<br>PF3D7_0937600/<br>PF3D7_0600400	1.55575	2.162734
PFA0765c	PF3D7_0115700	-0.5661	-1.91537
PFB1055c	PF3D7_0223500	-0.1358	-0.02741
PFC1120c/PFC0005w	PF3D7_0324900/PF3D7_0300100	-0.0349	-0.02741
PFD0005w	PF3D7_0400100	-0.2711	**-2.04335**
PFD0020c	PF3D7_0400400	0.0756	0.308632
PFD0615c	PF3D7_0412400	0.10955	0.240753
PFD0625c	PF3D7_0412700	0.9238	0.807795
PFD0630c/PFD0635c	PF3D7_0412900/PF3D7_0413100	1.04145	0.408632
PFD0995c	PF3D7_0420700	0.03815	0.114557
PFD1000c	PF3D7_0420900	3.4116	2.784209
PFD1005c	PF3D7_0421100	-0.1147	1.813794
PFD1015c	PF3D7_0421300	0.88525	1.580245
PFD1235w	PF3D7_0425800	0.28195	0.548978
PFD1245c	PF3D7_0426000	-0.0819	0.033889

PFE0005w	PF3D7_0500100	-0.10855	-0.03274
PFE1640w	PF3D7_0533100	-0.03585	-0.24163
PFI0005w	PF3D7_0900100	-0.0225	0.002329
PFI1820w	PF3D7_0937600	-0.30685	0.02691
PFI1830c	PF3D7_0937800	-0.71115	-0.72655
PFL0005w	PF3D7_1200100	0.0299	0.280334
PFL0020w	PF3D7_1200400	0.1116	0.423175
PFL0030c	PF3D7_1200600	-0.35135	-0.08584
PFL0935c	PF3D7_1219300	0.06835	0.118127
PFL1950w	PF3D7_1240300	0.0882	0.455534
PFL1955w/PFL1970w	PF3D7_1240400/PF3D7_1240900	0.99795	1.629812
PFL1960w	PF3D7_1240600	-0.08075	-1.08739
PFL2665c	PF3D7_1255200	-0.8997	-0.22437
